# Novel Iridoid Derivatives Isolated from the Roots of *Patrinia scabra* with Potential Anti-Renal Fibrosis Activity In Vitro

**DOI:** 10.3390/molecules29184419

**Published:** 2024-09-17

**Authors:** Ziran Li, Yang Xu, Xu Sun, Zhangrui Fan, Ziling Zhou, Fucai Ren, Ning Li, Lei Di

**Affiliations:** Anhui Provincial Laboratory of Inflammatory and Immunity Disease, Anhui Institute of Innovative Drugs, School of Pharmacy, Anhui Medical University, Hefei 230032, China

**Keywords:** *Patrinia scabra* Bunge, isovalerate, iridoid derivatives, renal fibrosis

## Abstract

Scabrol B and Scabrol C, two newly identified iridoid derivatives (**1** and **2**) and six known compounds (**3**–**8**), were extracted from the roots of *Patrinia scabra*. The structures of these derivatives, including their absolute configurations, were elucidated via comprehensive NMR analysis, chemical derivatization, and quantum chemical ECD calculations. All isolated compounds were evaluated for their anti-renal fibrosis activity. The results demonstrate that compounds **1** and **2** showed dose-dependent protective effects against renal fibrosis in vitro by reducing the expression of fibronectin, collagen I, and alpha-smooth muscle actin (*α*-SMA) in NRK-49f cells mediated by TGF-*β*1.

## 1. Introduction

*Patrinia scabra* Bunge is a perennial herb of the *Valerianaceae* family. Their roots have been used in traditional Chinese medicine (TCM) to treat malaria, dysentery, leukemia, stomach cancer, typhus, fall injuries, and leukorrhea [1,2]. Previous studies have indicated that *P. scabra* Bunge contains different secondary metabolites, the most common are iridoids [1,2,3,4], lignans [5], sesquiterpenes [6], triterpenoids [7], coumarins, and flavonoids [8]. Pharmacological studies have shown that the secondary metabolites of *Patrinia scabra* Bunge have antitumor, immunomodulatory, and anti-inflammatory activities [9,10,11]. However, its anti-renal fibrosis activity has not been widely reported. Meanwhile, it has been reported in the literature that iridoids from other medicinal plants play a nephroprotective role in acute kidney injury and diabetic nephropathy through anti-inflammatory activity [12,13,14].

In the present study, the chemical analysis of a 95% aqueous EtOH extract led to the isolation and characterization of two new isovaleryl iridoids (**1**–**2**) and six known compounds. The structures of the known compounds were determined by analyzing their spectroscopic data, including ^1^H NMR, ^13^C NMR, and HR-MS; the resultant data were compared with the reported literature. Moreover, a preliminary in vitro screening was performed using TGF-*β*1-induced kidney fibrosis in NRK-49f cells. Compounds **1** and **2** showed dose-dependent protective effects on reducing the expression of fibronectin, collagen I, and alpha-smooth muscle actin (α-SMA). Since fibronectin, collagen I, and α-SMA production is a main hallmark of renal fibrosis [15], this suggests that **1** and **2** might have anti-renal fibrosis effects. This finding is a part of the current project to identify new plant-derived anti-renal fibrosis agents. The current study showed the isolation, structural elucidation, and inhibitory activity of the novel iridoid derivatives against kidney fibrosis.

## 2. Results and Discussion

### 2.1. Structure Elucidation of the Compounds

**Figure 1:**
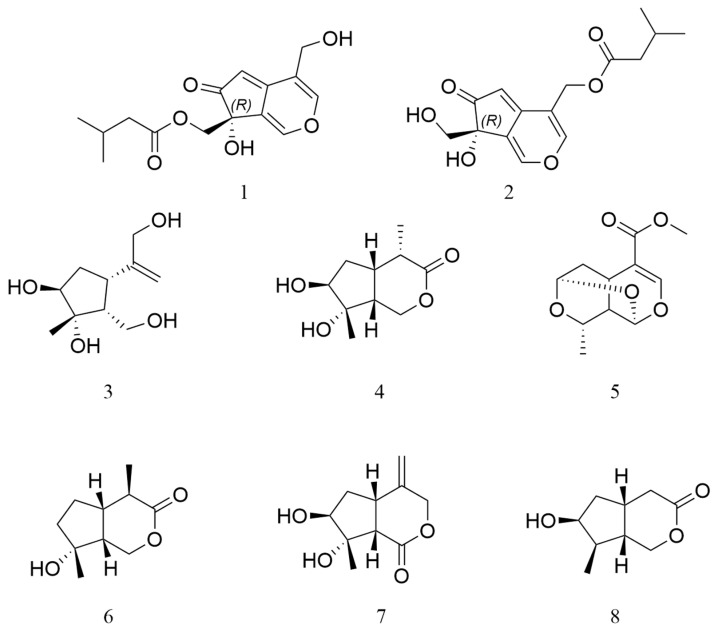
Structures of compounds **1**–**8** from Patrinia scabra Bunge.

A 95% aqueous EtOH extract of the roots of *P. scabra* (20 kg) was extracted with petroleum ether, ethyl acetate, dichloromethane, and n-butanol. The EtOAc layers were subjected repeatedly to column chromatography to afford two new iridoid derivatives (**1** and **2**) and six known compounds (Figure 1).

**Figure 2:**
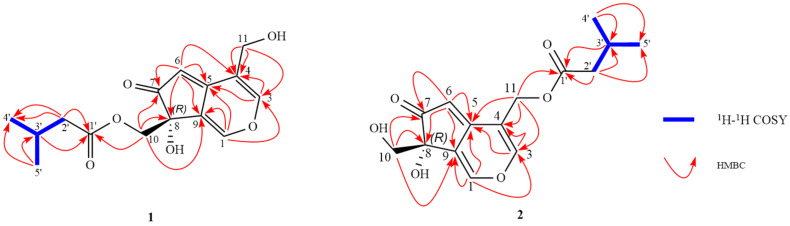
Key ^1^H-^1^H COSY and HMBC correlations of **1**–**2**.

**Figure 3:**
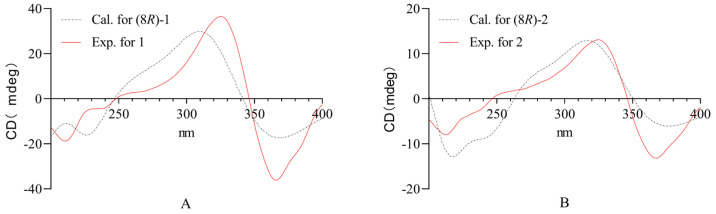
Comparison of the calculated ECD and experimental CD spectra in MeOH. (**A**) Calculated ECD spectrum of (8*R*)-**1** at B3LYP/6-311+g (2d, p) level, =0.20 eV; shift = +9 nm. (**B**) Calculated ECD spectrum of (8*R*)-**2** at B3LYP/6-311+g (2d, p) level, =0.30 eV; shift = +9 nm.

Compound **1**, isolated as colorless gums, has the molecular formula of C_15_H_18_O_6_ (7 degrees of unsaturation) based on the analysis of the HR-MS in the positive ion mode, *m*/*z* 317.1022 [M + Na]^+^ (calcd for C_15_H_18_O_6_Na, 317.1001) (Figure S6). The ^1^H NMR spectrum of **1** (Table 1, Figure S1) shows characteristic signals of iridoid derivatives, such as two methyl groups [*δ*_H_ 0.90 (3H, d, *J* = 1.88 Hz, H-4′), 0.91 (3H, d, *J* = 1.88 Hz, H-5′)], three methylenes [*δ*_H_ 4.39 (1H, d, *J* = 10.90 Hz, Ha-10), 4.04 (1H, d, *J* = 10.92 Hz, Hb-10), 4.38 (2H, s, H-11), 2.18 (2H, d, *J* = 7.16 Hz, H-2′)], and four methines [*δ*_H_ 7.46 (1H, s, H-1), 7.53 (1H, s, H-3), 5.44 (1H, s, H-6), 2.00 (1H, m, H-3′]. The ^13^C NMR, DEPT spectra, and the heteronuclear single quantum coherence spectroscopy (HSQC) spectra (Table 1, Figure S2) revealed the presence of two methyls [*δ*_C_ 22.60 (C-4′), 22.60 (C-5′)], one methylene [*δ*_C_ 43.91 (C-2′)], two oxygenated methylenes [*δ*_C_ 66.72 (C-10), 59.06 (C-11)], two methines [*δ*_C_ 106.06 (C-6), 26.79 (C-3′)], two oxygenated olefinic methines [*δ*_C_ 141.93 (C-1), 150.53 (C-3)], four quaternary carbon [*δ*_C_ 120.64 (C-4), 161.20 (C-5), 75.32 (C-8), 129.62 (C-9)], and two carbonyl groups [*δ*_C_ 204.88 (C-7), 173.98 (C-1′)]. Correlation spectroscopy (COSY) was used to determine the associations between H3′/4′, H3′/5′, and H3′/2′ and the existence of the isovalerate group by analyzing the HMBC (Figure 2) correlations of H5′/C3, C4′, H3′/C1′, H2′/C1′, C3′, and C4′, as well as the chemical shift of C-1′ (δ_C_ 173.98). The study found that there were heteronuclear multiple bond coherence (HMBC) correlations between H1/C3, C9 (*δ*_C_ 129.62), H3/C1, C4 (*δ*_C_ 120.64), C5 (*δ*_C_ 161.2), H6/C5, C7 (*δ*_C_ 204.88), C8 (*δ*_C_ 75.32), C9, C4, H11/C3, C4, C5, H10/C7, C8, and C9. These assignments were consistent with the iridoid structure of **1**. The iridoid structure and the isovalerate group were connected between C-1′ and C-10 via an O-linkage from the correlation from *δ*_H_ 4.39 (Ha-10) and 4.04 (Hb-10) to C-1′ (*δ*_C_ 173.98). The configuration of 1 was confirmed by ECD calculations (Figure 3A). This experimental spectrum was similar to the calculated 8*R*-**1**. Based on these results, compound **1** was designated as Scabrol B.

Compound **2** has the molecular formula of C_15_H_18_O_6_ from its HR-MS (*m*/*z* 317.0953 [M + Na]^+^, (calcd for C_15_H_18_O_6_Na, 317.1001)) (Figure S12), indicating the presence of 7 degrees of unsaturation. The ^13^C-NMR spectra of 1 and 2 were similar, except that, in compound **1**, the isovaleryl group was linked at C10, whereas in compound **2**, the isovaleryl group (*δ*_C_ 22.65, 26.91, 43.96, and 174.24) was linked at C11 (*δ*_C_ 61.09). The iridoid structure and the isovalerate group were connected between C-1′ and C-11 via an O-linkage from the correlation from *δ*_H_ 3.70 (Ha-11) and 3.54 (Hb-11) to C-1′ (*δ*_C_ 172.24). The absolute configuration of compound 2 was verified via quantum chemical electronic circular dichroism (ECD) calculations (Figure 3B). The experimental ECD spectrum of compound **2** showed a significant resemblance to the calculated spectrum for the 8*R* configuration of the compound. Combining these results with the existing literature, compound **2** was identified as Scabrol C.

All known chemical compounds were identified as Jatamanin J (**3**) [8], Isopatriscabrol (**4**) [16], sarracenin (**5**) [17], scabrol A (**6**) [4], Jatamanin A (**7**) [8], and Isoboonein (**8**) [18]. Their identification was performed by comparing their spectroscopic data with data published in the relevant literature.

### 2.2. Biological Evaluation

**Figure 4:**
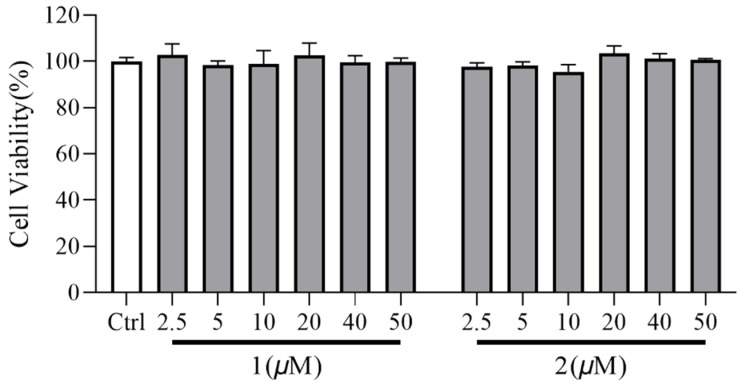
NRK-49f cell proliferation in response to compounds **1** and **2** by CCK-8 assay.

**Figure 5:**
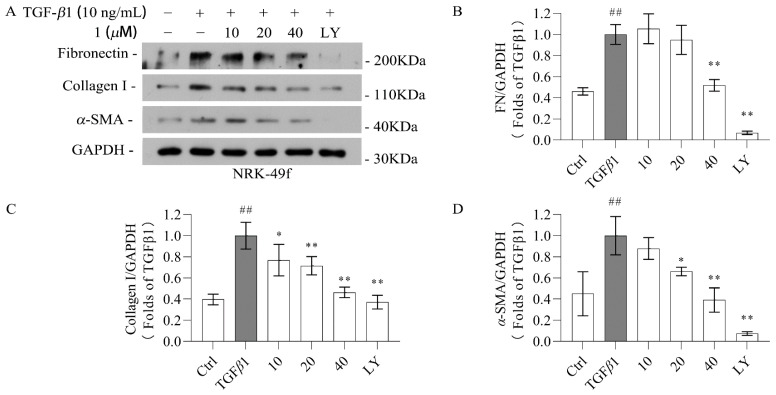
Compound **1** inhibits kidney fibrosis in cells induced by TGF-*β*1. Cells were treated with different doses of the compound and then incubated with 10 ng/mL TGF-*β*1 for 48 h. (**A**–**D**) Western blotting was used to determine the protein levels of fibronectin, collagen I, and α-SMA in NRK-49f. GAPDH was used as a control. Data were represented as the mean ± SEM. * *p* < 0.05, ** *p* < 0.01 vs. the TGF-β1 group. ## *p* < 0.01 vs. the Ctrl group. Galunisertib (LY) was used as a positive control.

**Figure 6:**
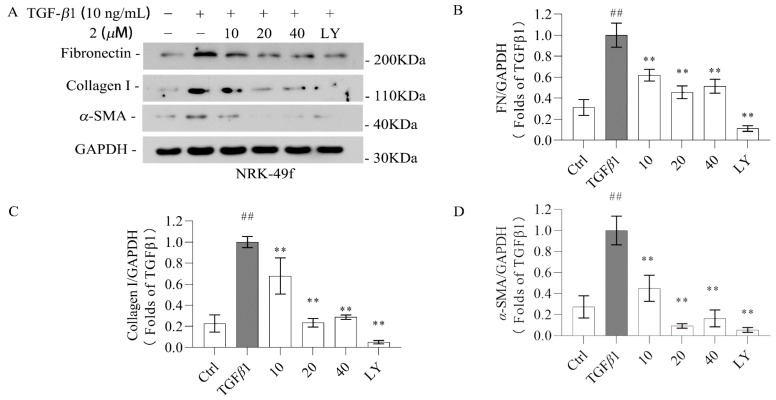
Compound **2** inhibits kidney fibrosis in cells induced by TGF-*β*1. Cells were treated with different doses of the compound and then incubated with 10 ng/mL TGF-*β*1 for 48 h. (**A**–**D**) Western blotting was used to determine the protein levels of fibronectin, collagen I, and α-SMA in NRK-49f. GAPDH was used as a control. Data were represented as the mean ± SEM. ** *p* < 0.01 vs. the TGF-β1 group. ## *p* < 0.01 vs. the Ctrl group. Galunisertib (LY) was used as a positive control.

The effect of novel compounds **1** and **2** on anti-renal fibrosis was investigated in TGF-β1-induced NRK-49f cells. To establish that the biological effects of compounds **1**–**8** are not due to cytotoxicity, a CCK-8 assay was conducted (Figure 4; Figure S15). The results show that compounds **1** and **2** were not toxic to NRK-49f cells at 40 µM. The other compounds were selected at the maximum non-toxic dose for subsequent activity screening and the initial screening showed that compounds **1** and **2** exhibited the best activity. Thus, the renal protective effect of compounds 1 and 2 was further evaluated. Both of these compounds decreased the expression of fibronectin, collagen I, and α-SMA in TGF-*β*1-induced NRK-49f cells in a dose-dependent manner, as evidenced by the data in Figure 5 and Figure 6. Compounds **3**–**8** did not show anti-fibrotic effects (Figure S16). Considering that fibronectin, collagen I, and α-SMA are key components of the extracellular matrix (ECM), and the overexpression of the ECM is recognized as a critical marker of renal fibrosis, the present findings indicate that iridoid derivatives could be promising agents for renal protection.

## 3. Materials and Methods

### 3.1. General Procedures, Materials, and Reagents

Optical rotations were recorded on an Anton Par MCP-150 digital polarimeter. The spectra of CD were assessed using a Chirascan instrument (Agilent Technologies, Santa Clara, CA, USA). The NMR spectra were collected by a Bruker Avance III 600 MHz spectrometer (Billerica, MA, USA), and the internal standard was TMS. HRESIMS were recorded on a Shimazu LC-20AD AB Sciex triple X500R MS spectrometer (Shimadzu Corporation, Tokyo, Japan).

Chromatographic materials included: MCI gel CHP 20P (75–150 µm, Mitsubishi Chemical Industries, Tokyo, Japan), Biotage^®^ Sfär C18 Duo (30 µm; Bitotage, Uppsala, Sweden), Sephadex LH-20 (Psaitong Biotechnology, Beijing, China), and Gel column chromatography was performed on silica gel (300–400 mesh, Qingdao Marine Chemical Inc., Qingdao, China). A Thermos Fisher Scientific Ultimate 3000 HPLC system (Waltham, MA, USA) was used for semi-preparative HPLC with a Thermo Hypersil GOLD-C18 column (Waltham, MA, USA) (250 mm × 10 mm, i.d., 5 µm).

The liquid reagents used in all experiments contained: CH_3_OH (AR, G75851E, Greagent, Shanghai, China); Ethyl Acetate (AR, G23272G, Greagent, Shanghai, China); Dichloromethane (AR, G81014H, Greagent, Shanghai, China); Petroleum Ether (AR, G84208Iy, Greagent, Shanghai, China); 1-Butanol (AR, G78625G, Greagent, Shanghai, China); 95% Ethanol (G73537AN, Greagent, Shanghai, China); Methanol (gradient HPLC, A040747, Energy chemical, Shanghai, China); DMSO (sterile-filtered ≥ 99.7%, D2650, Sigma-Aldrich, Darmstadt, Germany); and Methanol-d3 (M276588-25g, Energy chemical, Shanghai, China).

### 3.2. Plant Material

*Patrinia scabra* was purchased from Bozhou, Anhui Province, China, in May 2020 and identified by Prof. Chuan-pu Shen from the School of Pharmacy, Anhui Medical University, China. A voucher specimen (MTH-202005) was submitted to the School of Pharmacy, Anhui Medical University, China.

### 3.3. Extraction and Isolation

The air-dried powdered roots of *P. scabra*. (20 kg) were extracted with 95% EtOH (3 × 75 L, 24 h each) at room temperature. A crude extract (3.3 kg) was prepared by concentrating the initial material and then sequentially extracting it with petroleum ether, ethyl acetate, dichloromethane, and n-butanol. The solvents were then removed under reduced pressure to yield fractions of different polarities, which were designated as Fr.1 (221 g), Fr.2 (108 g), Fr.3 (120 g), Fr.4 (1.2 kg), and Fr.5 (651 g). Fr.3 (120 g) was isolated by silica gel columns with CH_2_Cl_2_:MeOH (100:0–10:1) to produce 8 fractions (Fr.3-1–Fr.3-8). Fr.3–2 (6.9 g) was fractionated via Sephadex LH-20 (MeOH:H_2_O = 50:50–90:10) resulting in 4 fractions (Fr. 3-2-1–Fr. 3-2-4). Fr. 3-2-1 (3.5 g) was purified by MCI Gel CHP-20 (MeOH:H_2_O = 10:90–100:0) to yield 17 portions (Fr. 3-2-1-1–Fr. 3-2-1-17). Fr. 3-2-1-12 (70.0 mg) was purified by semi-preparative HPLC to obtain compound **5** (9 mg). Fr. 3-4 (20 g) was fractionated to MCI Gel CHP-20 (MeOH:H_2_O = 10:90–80:20) to produce 18 fractions (Fr.3-4-1–Fr. 3-4-18). Fr. 3-4-1 (20 g) was separated by MCI Gel CHP-20 (MeOH:H_2_O = 10:90–30:70) to yield 4 portions (Fr. 3-4-1-4–Fr. 3-4-1-4). Fr. 3-4-1-1 was recrystallized to obtain compound **4** (8 g). Fr. 3-3 (7.4 g) was separated into MCI Gel CHP-20 (MeOH:H_2_O = 10:90–100:0) to yield 17 portions (Fr. 3-3-1–Fr. 3-3-7). Fr. 3-3-1 (3.1 g) was purified by C18 column on an MPLC system eluted with gradient aqueous (MeOH:H_2_O = 10:90–100:0, flow rate: 35 mL/min) to obtain 2 portions (Fr. 3-3-1-1–Fr. 3-3-1-2). Fr. 3-3-1-1 (1.1 g) was separated into 3 fractions (Fr. 3-3-1-1-1–Fr. 3-3-1-1-3) by Sephadex LH-20 column chromatography (aqueous MeOH, 100%). Fr. 3-3-1-1-1 (300 mg) was fractionated to silica gel columns on an MPLC system with CH_2_Cl_2_:MeOH (20:1) to produce compound **8** (8 mg). Fr. 3-3-1-1-2 (300 mg) was separated into silica gel columns on an MPLC system with CH_2_Cl_2_:MeOH (30:1) to produce 3 fractions (Fr. 3-3-1-1-2-1, Fr. 3-3-1-1-2-2 (compound **7** (14.8 mg)), Fr. 3-3-1-1-2-3). Fr. 3-3-1-1-2-1 (85 mg) was separated by preparative HPLC to obtain compound **6** (1.2 mg). Fr. 3-5 (31 g) was separated into 9 fractions (Fr. 3-5-1–Fr. 3-5-9) by MCI Gel CHP-20 columns (aqueous MeOH:H_2_O = 10:90–100:0). Fr. 3-5-5 (2.8 g) was separated by Sephadex LH-20 column chromatography (MeOH:H_2_O = 10:90–100:0) to yield 10 portions (Fr. 3-5-5-1–Fr. 3-5-5-10). Fr. 3-5-5-2 (520 mg) was purified by C18 column on an MPLC system eluted with gradient aqueous (MeOH:H_2_O = 10:90, flow rate: 35 mL/min) to obtain Fr. 3-5-5-2-1 and Fr. 3-5-5-2-2. Fr. 3-5-5-2-2 (210 mg) was purified to obtain compound **1** (20 mg), compound **2** (5 mg), and compound **3** (70 mg).

### 3.4. Computational Methods

Quantum chemical calculations were performed via Gaussian 16 software. The GMMX module is used for searching the compound conformations. Electronic circular dichroism (ECD) calculations were carried out at the B3LYP/6-311g + (2d, p) level, and the resulting CD spectra were generated via the SpecDis 1.62 program (Figures S13 and S14).

### 3.5. Anti-Fibrosis Activity

#### 3.5.1. Cell Culture

NRK-49f, a cell line that showed fibroblast morphology (Cell Resource Center, IBMS, CAMS/PUMC), was cultured in DMEM/F12 (C11330500BT, Gibco, Waltham, MA, USA) enriched with 10% fetal bovine serum (FBS) (RY-F22, Royacel, Lanzhou, China), and 1% penicillin/streptomycin (KeyGEN Biotech, Nanjing, China) at 37 °C in a humidified environment with 5% CO_2_.

#### 3.5.2. Cell Viability Assay

A total of 1 × 10⁴ cells/mL (NRK-49f cells) were seeded into a 96-well plate containing complete DMEM/F12. After 24 h incubation, the cells were treated with compounds **1**–**8** with a gradient concentration of at most of maximum non-toxic dose or dimethyl sulfoxide (DMSO) for 48 h. Next, 10 µL Cell Count Kit-8 (CCK-8, K1018, APExBIO, Houston, TX, USA) was added to each well and incubated for 1.5 h at 37 °C. The absorbance of each well was measured at 450 nm using a microplate reader (SpectraMax iD3, Molecular Devices, Shanghai, China).

#### 3.5.3. Western Blotting

NRK-49f cells were incubated with 10 ng/mL TGF-*β*1 (Peprotech, 100-21, Waltham, MA, USA) for 48 h with or without different concentrations of compounds. Cells were lysed with RIPA buffer (Servicebio, G2002, Wuhan, China) containing 1× protease inhibitor cocktail (Yeasen Biotechnology, 20124ES03, Shanghai, China) and 1× phosphatase inhibitor cocktails (Yeasen Biotechnology, 20109ES05, Shanghai, China) with 0.1 mM PMSF. Proteins were quantified via the BCA assay (Servicebio, G2026, Wuhan, China). Equal quantities of protein extracts were loaded to SDS-PAGE (8–10% gel) and then transferred to nitrocellulose (NC) membranes (0.45 µM Millipore, Germany). The membranes were blocked with 5% BSA for 1 h, and then incubated with the respective primary antibodies overnight at 4 °C. After incubation with HRP-conjugated secondary antibodies at ambient temperature, the bands were visualized and measured using the ECL equipment (Applygen, P1050, Beijing, China) [19]. Primary antibodies include fibronectin polyclonal antibody (Proteintech, 15613-1-AP, Wuhan, China), collagen Type I/COL1A1 antibody (Boster, BM0325, Wuhan, China), α-SMA monoclonal antibody (Abcam, ab7817, Cambridge, UK), and GAPDH monoclonal antibody (Proteintech, 60004-1-Ig, Wuhan, China).

## 4. Conclusions

In conclusion, this study identified novel iridoid derivatives from the roots of *P. scabra*, expanding the structural diversity of iridoids. Biological assays demonstrated that iridoid derivatives with an isovalerate group at C-10 or C-11 can inhibit kidney fibrosis in vitro. To the best of our knowledge, there are limited reports on iridoid derivatives with the isovalerate group against renal fibrosis. In the structural relationship studies, the effect of the isovaleryl group was assessed on the anti-renal fibrosis activity of iridoids. The results show that compounds **1** and **2** (at non-toxic doses) had significantly higher anti-fibrotic activity compared to other compounds without the isovaleryl group. However, due to the limited number of compounds and the absence of additional isovaleryl iridoids, further research with a larger sample size is needed to fully elucidate the activity. Moreover, additional research is necessary to understand the impact of the isovaleryl group on the bioactivity of iridoid derivatives from *P. scabra* Bunge, especially concerning anti-renal fibrosis. More studies are also needed to clarify the underlying molecular mechanisms. Lastly, exploring the anti-fibrotic activity of isovalerylated iridoid derivatives could provide valuable insights into the biological profiling of compounds from *P. scabra* Bunge.

## Figures and Tables

**Table 1:** Data of ^1^H NMR (600 MHz) and ^13^C NMR (151 MHz) in CD_3_OD (δ in ppm, J in Hz) of **1**–**2**.

Position	^13^C NMR of 1	^13^C NMR of 2	^1^H NMR of 1	^1^H NMR of 2
1	141.93, CH	141.46, CH	7.46 (s)	7.44 (s)
3	150.53, CH	152.98, CH	7.53 (s)	7.64 (s)
4	120.64, C	116.03, C		
5	161.20, C	160.82, C		
6	106.06, CH	106.86, CH	5.44 (s)	5.41 (s)
7	204.88, C	206.36, C		
8	75.32, C	77.77, C		
9	129.62, C	130.48, C		
10	66.72, CH_2_	66.26, CH_2_	Ha: 4.39 (d, 10.90) Hb: 4.04 (d, 10.92)	Ha: 3.70 (d, 11.09) Hb: 3.54 (d, 11.12)
11	59.06, CH_2_	61.09, CH_2_	4.38 (s)	4.92 (s)
1′	173.98, C	174.24, C		
2′	43.91, CH_2_	43.96, CH_2_	2.18 (d, 7.16)	2.22 (m)
3′	26.79, CH	26.91, CH	2.00 (m)	2.06 (m)
4′	22.60, CH_3_	22.65, CH_3_	0.90 (d, 1.88, overlap)	0.94 (overlap)
5′	22.60, CH_3_	22.65, CH_3_	0.91 (d, 1.88, overlap)	0.94 (overlap)

## Data Availability

All the data in this research are presented in manuscript and Supplementary Materials.

## Supplementary Materials

The following are available online at https://www.mdpi.com/article/10.3390/molecules29184419/s1, Figures S1–S5: NMR spectra of compound **1**. Figure S6: HRESIMS of compound **1**. Figures S7–S11: NMR spectra of compound **2**. Figure S12: HRESIMS of compound **2**. Figures S13 and S14: Optimized geometries of predominant conformers for compounds **1**–**2**. Figure S15: NRK-49f cell proliferation in response to compounds **3**–**8**. Figure S16: Effect of **1**–**8** on the expression of fibronectin in TGF*β*1-induced NRK-49f.
