# Supplementary material for: Novel Iridoid Derivatives Isolated from the Roots of Patrinia scabra with Potential Anti-Renal Fibrosis Activity In Vitro
Source: Molecules. 2024 Sep 17;29(18):4419. doi: 10.3390/molecules29184419 (PMC11433686; doi:10.3390/molecules29184419)
Supplement: Supplementary file 1 [file molecules-29-04419-s001.zip › molecules-3201448-supplementary.pdf]

Supplementary Materials

Novel Iridoid Derivatives Isolated from the Roots of *Patrinia scabra* with Potential Anti-Renal Fibrosis Activity *in vitro*

Zi-ran Li[1], Yang Xu[1], Xu Sun[1], Zhang-rui Fan[1], Zi-ling Zhou[1], Fu-cai Ren[1], Ning Li[1*], Lei Di[1*†]

1 Inflammation and Immune Mediated Diseases Laboratory of Anhui Province, School of Pharmacy, Anhui Medical University, Hefei 230032, China

* Correspondence: 1993500019@ahmu.edu.cn (N. L.); dilei@ahmu.edu.cn (L. D.); Tel./Fax: +86-0551-65172133 (L. D.)

† Lead contact.

Contents

Supplementary Figures

Figure S1. ^{1}H NMR spectrum of 1 in CD_3OD
Figure S2. ^{13}C NMR and DEPT spectra of 1 in CD_3OD
Figure S3. HSQC spectrum of 1 in CD_3OD
Figure S4. HMBC spectrum of 1 in CD_3OD
Figure S5. ^{1}H-^{1}H COSY spectrum of 1 in CD_3OD
Figure S6. HRESIMS of 1
Figure S7. ^{1}H NMR spectrum of 2 in CD_3OD
Figure S8. ^{13}C NMR and DEPT spectra of 2 in CD_3OD
Figure S9. HSQC spectrum of 2 in CD_3OD
Figure S10. HMBC spectrum of 2 in CD_3OD
Figure S11. ^{1}H-^{1}H COSY spectrum of 2 in CD_3OD
Figure S12. HRESIMS of 2
Figure S13 Optimized geometries of predominant conformers for 1
Figure S14 Optimized geometries of predominant conformers for 2
Figure S15: NRK-49f cell proliferation in response to compounds 3-8.
Figure S16: Effect of 1-8 on the expression of fibronectin in TGFβ1 induced NRK-49f.

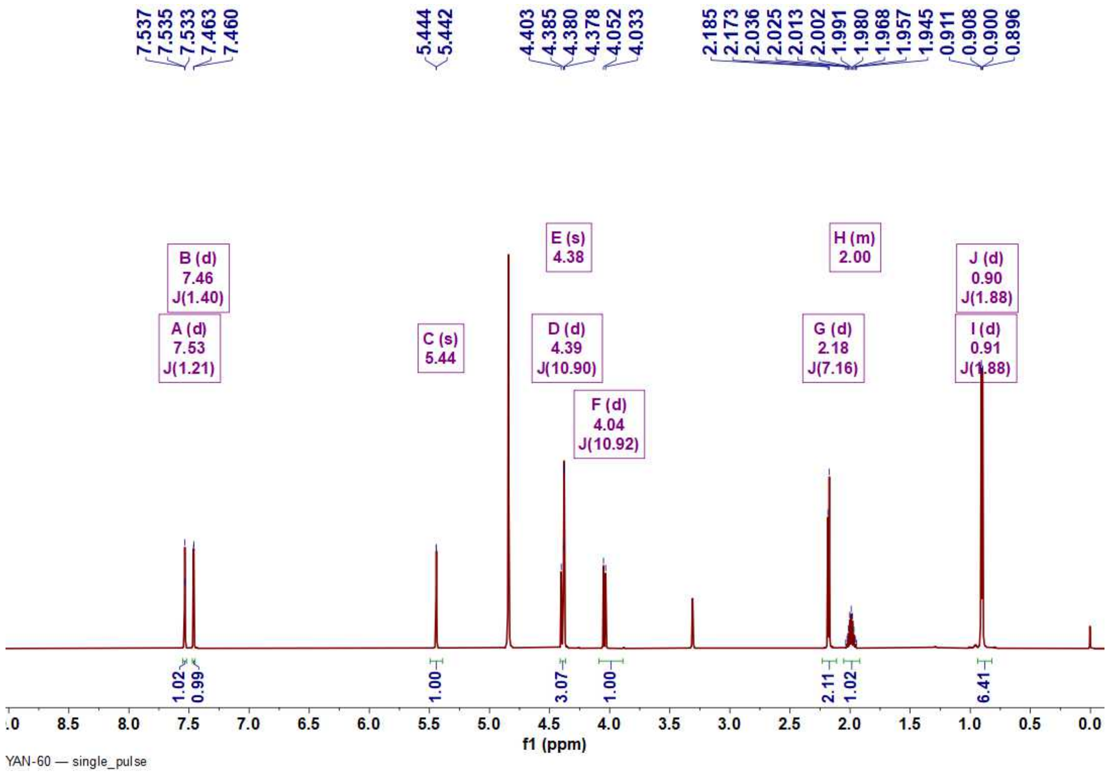

Figure S1. ^{1}H NMR spectrum of 1 in CD_3OD

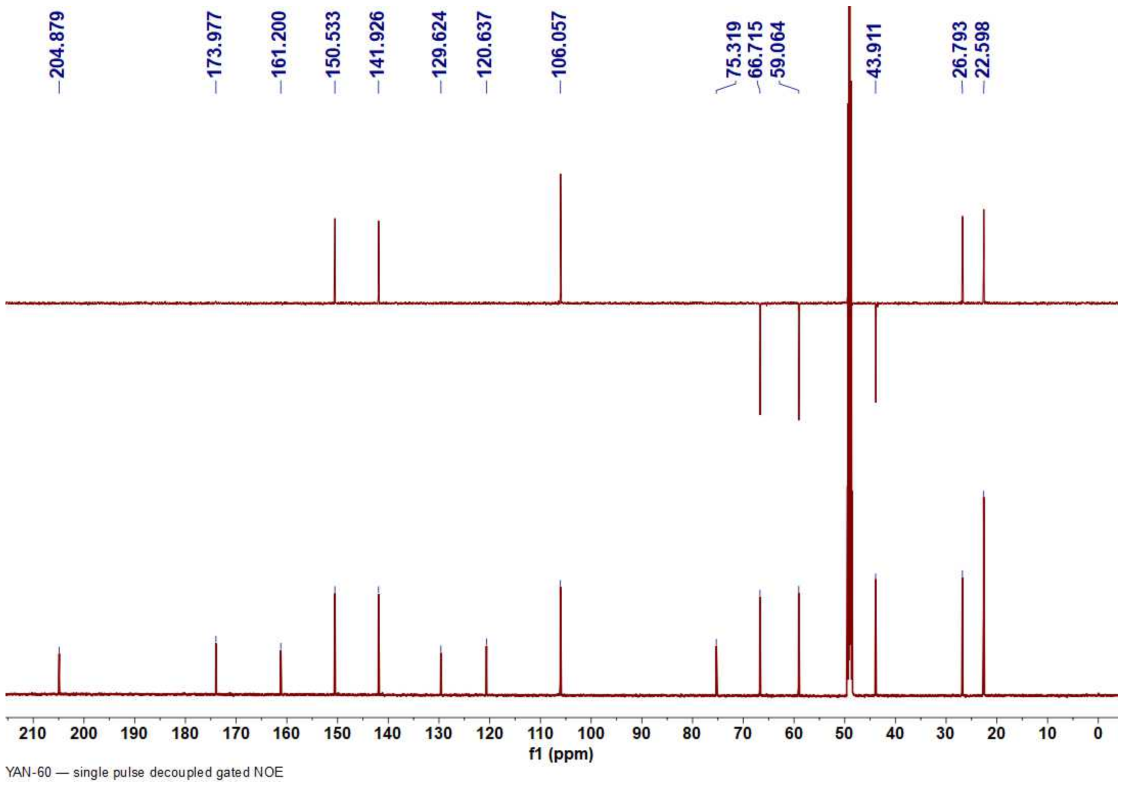

Figure S2. ^{13}C NMR and DEPT spectra of 1 in CD_3OD

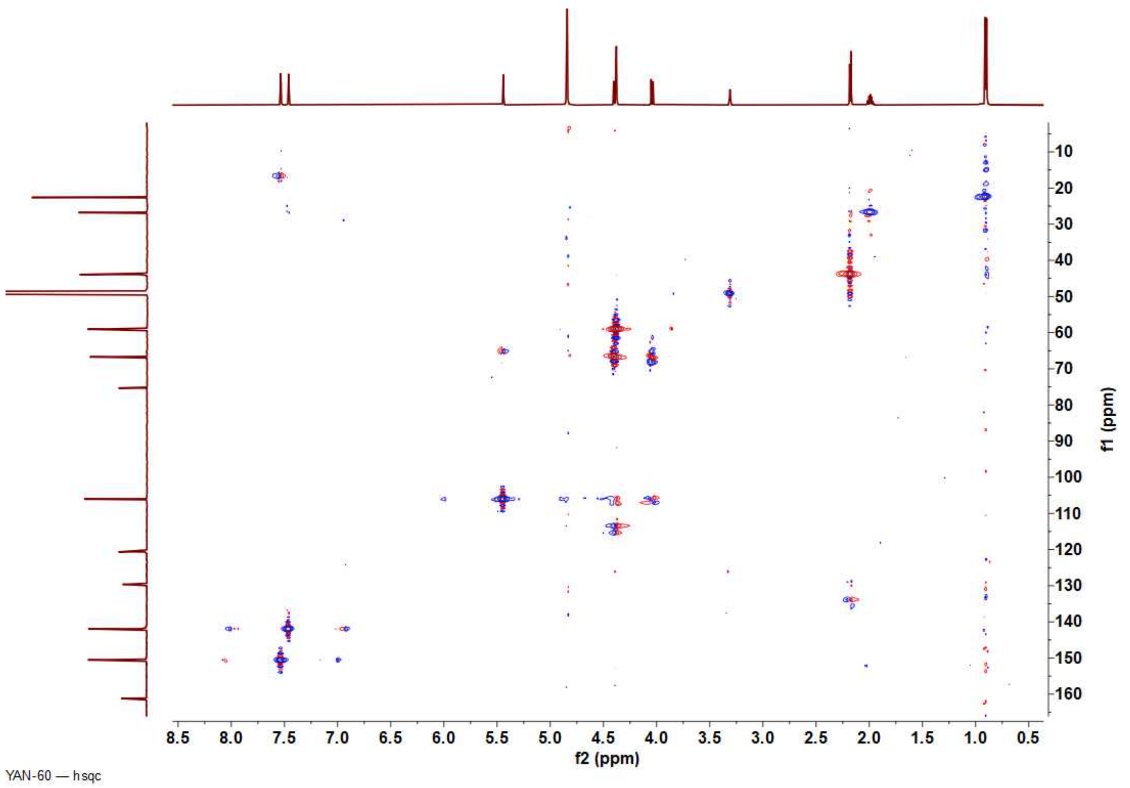

Figure S3. HSQC spectrum of 1 in CD_3OD

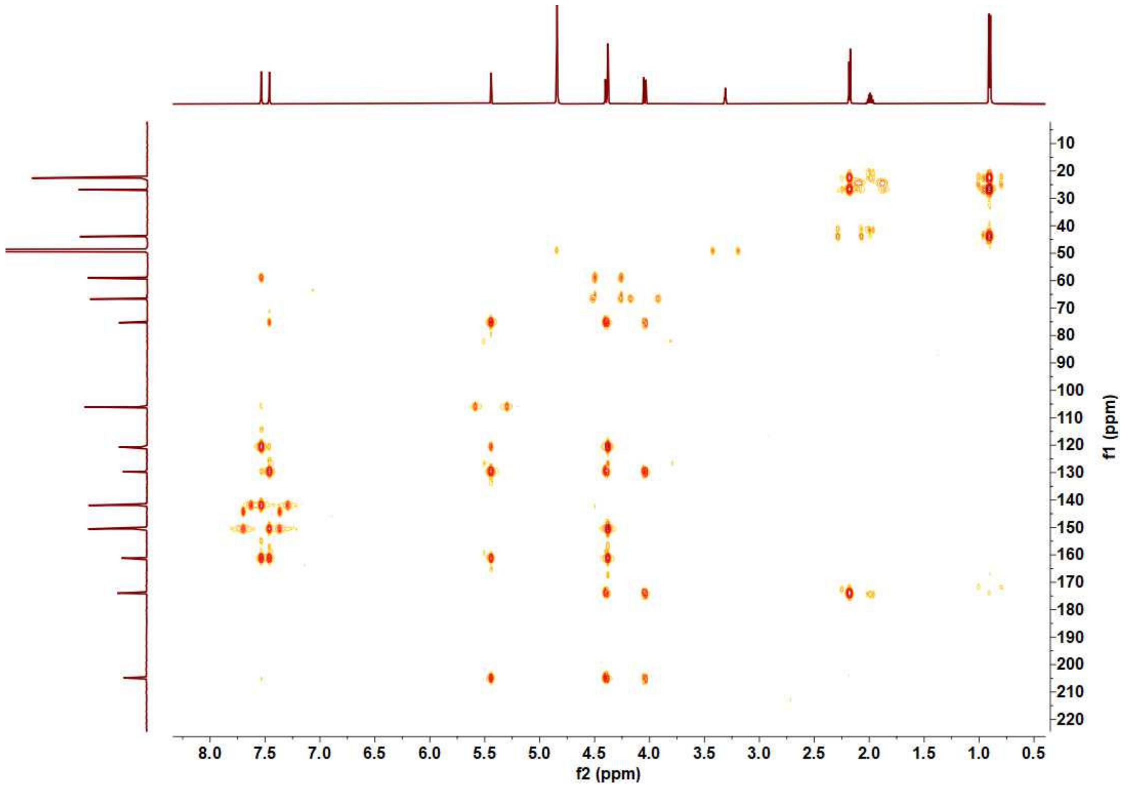

Figure S4. HMBC spectrum of 1 in CD_3OD

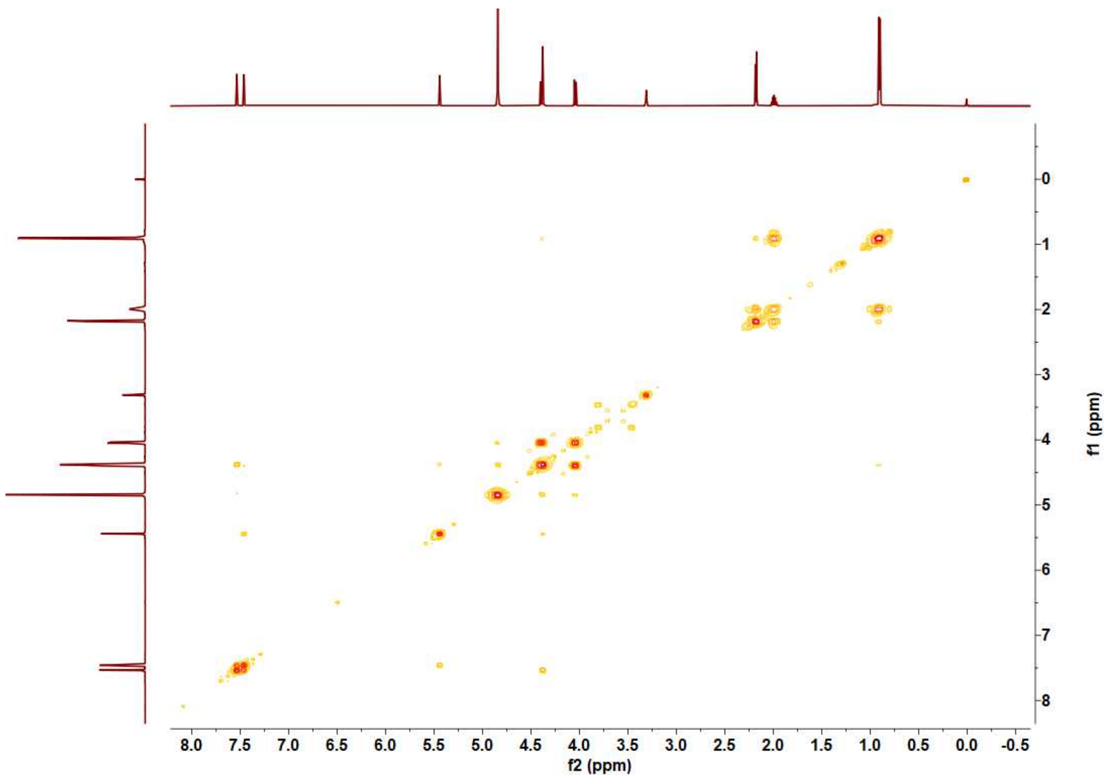

Figure S5. ^{1}H-^{1}H COSY spectrum of 1 in CD_3OD

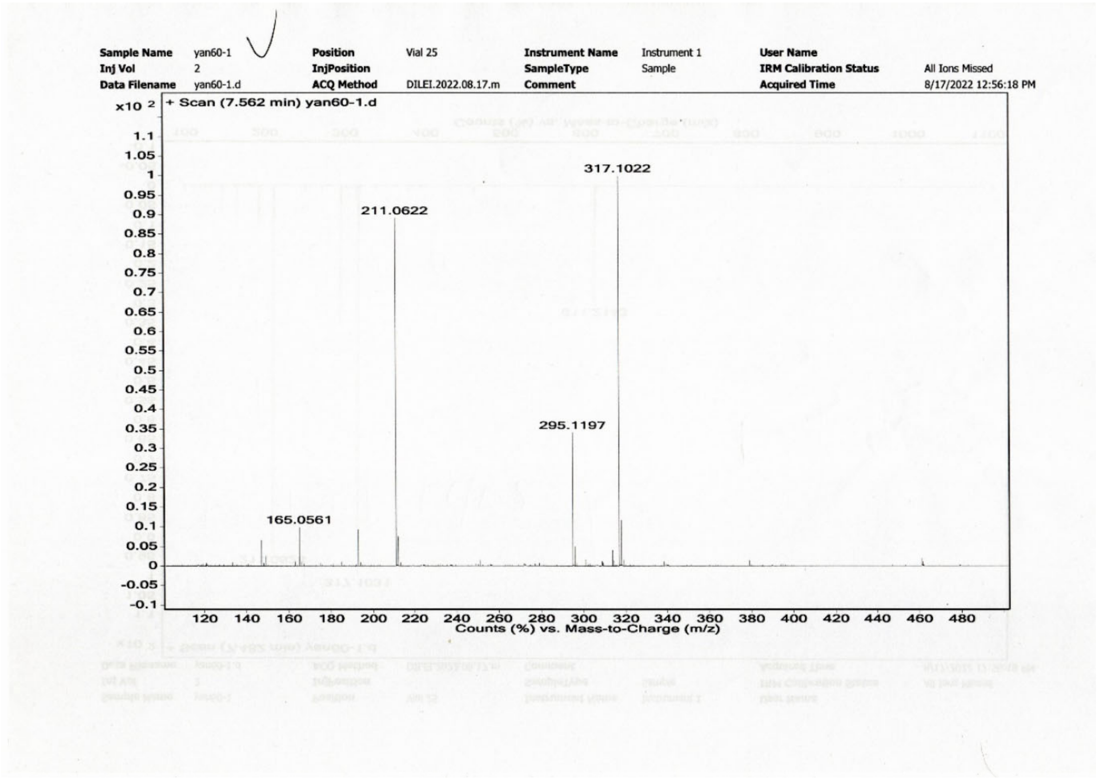

Figure S6. HRESIMS of 1

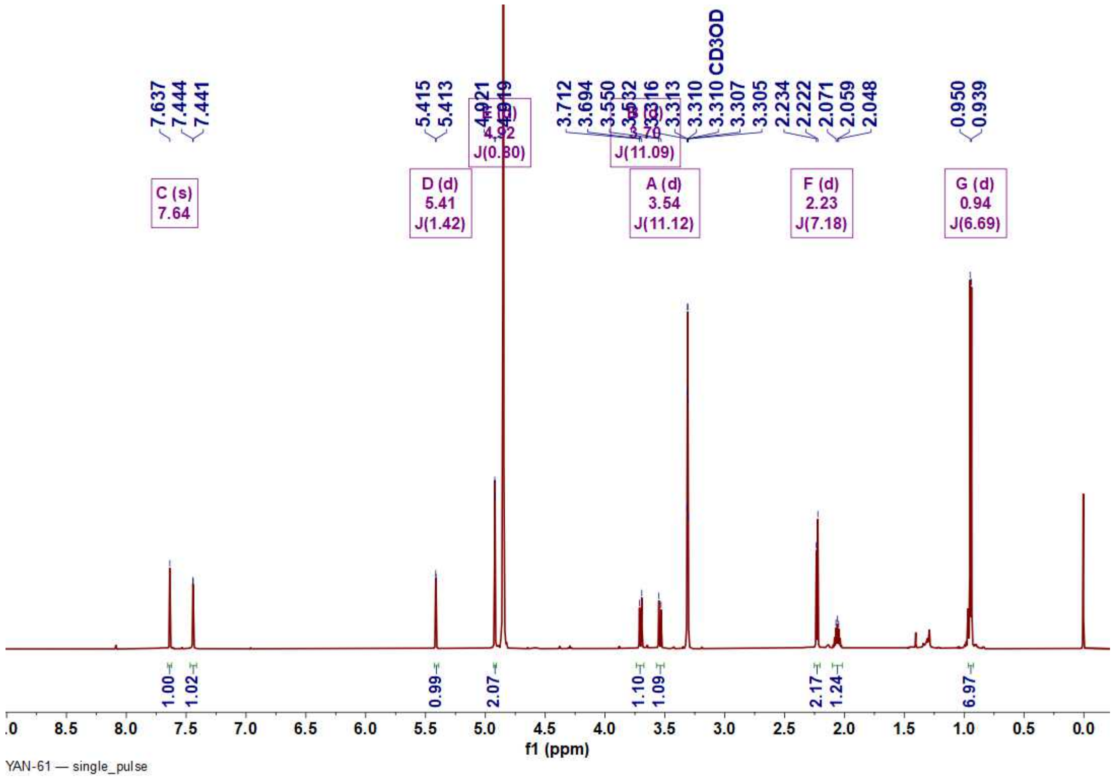

Figure S7. ^1H NMR spectrum of 2 in CD_3OD

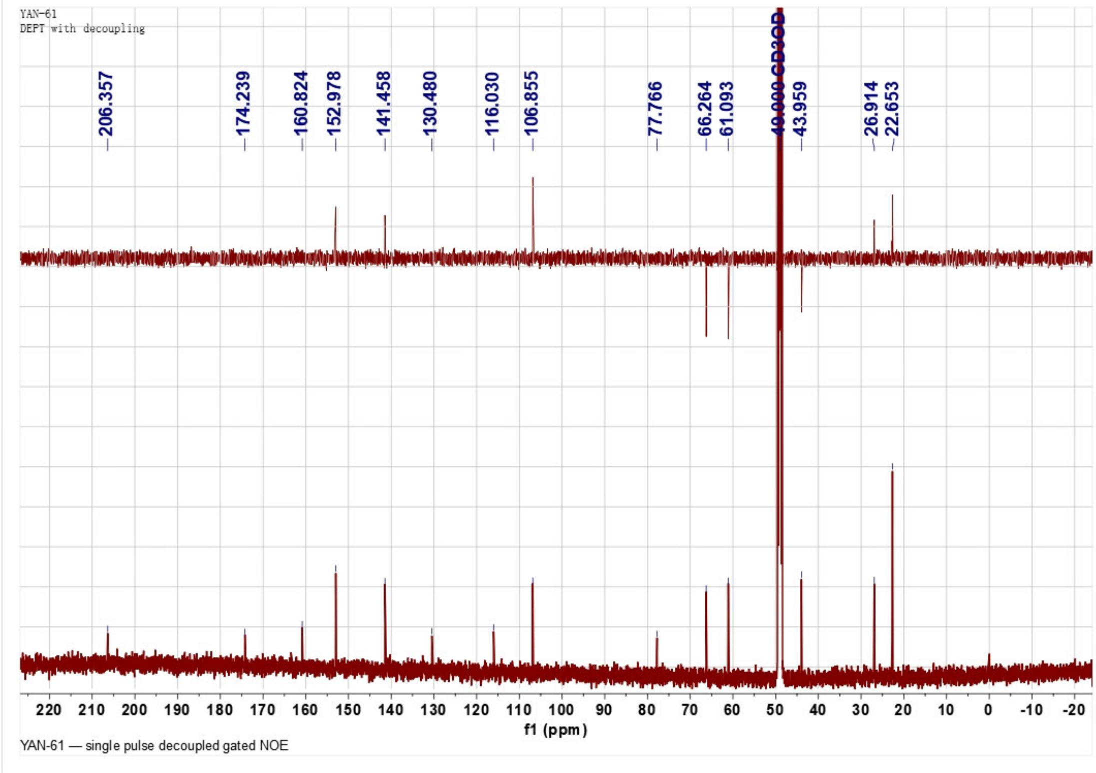

Figure S8. ^{13}C NMR and DEPT spectra of 2 in CD_3OD

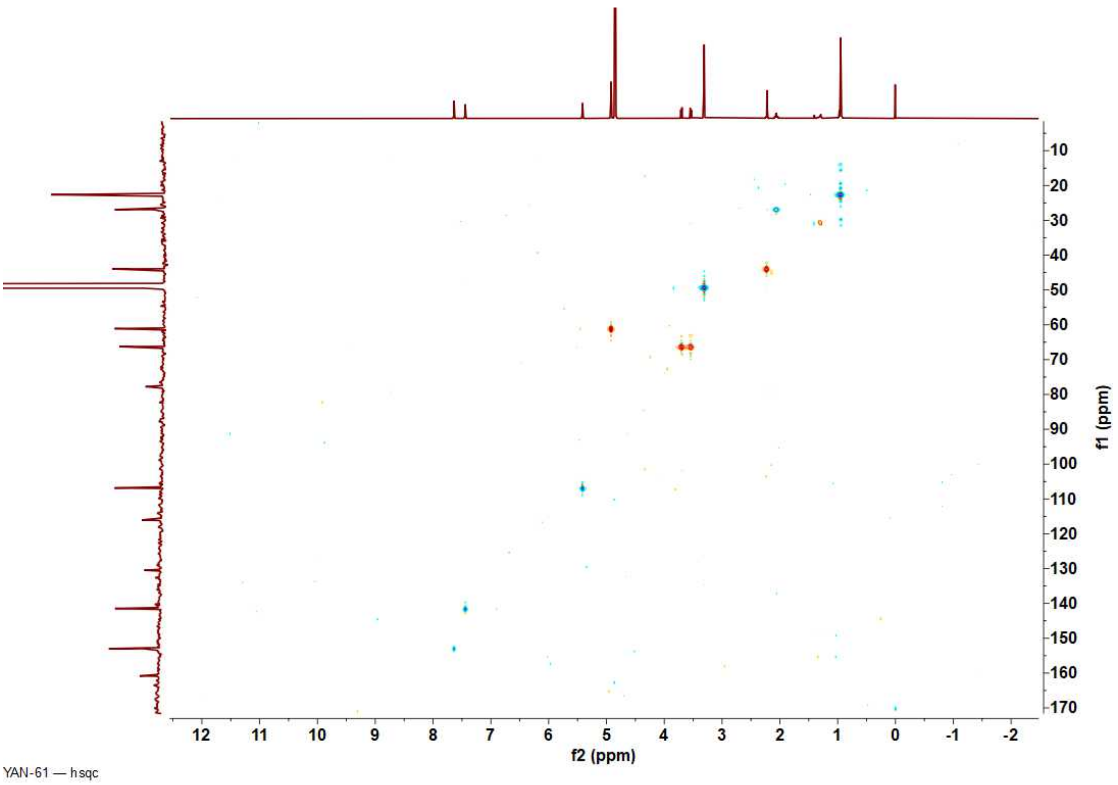

Figure S9. HSQC spectrum of 2 in CD_3OD

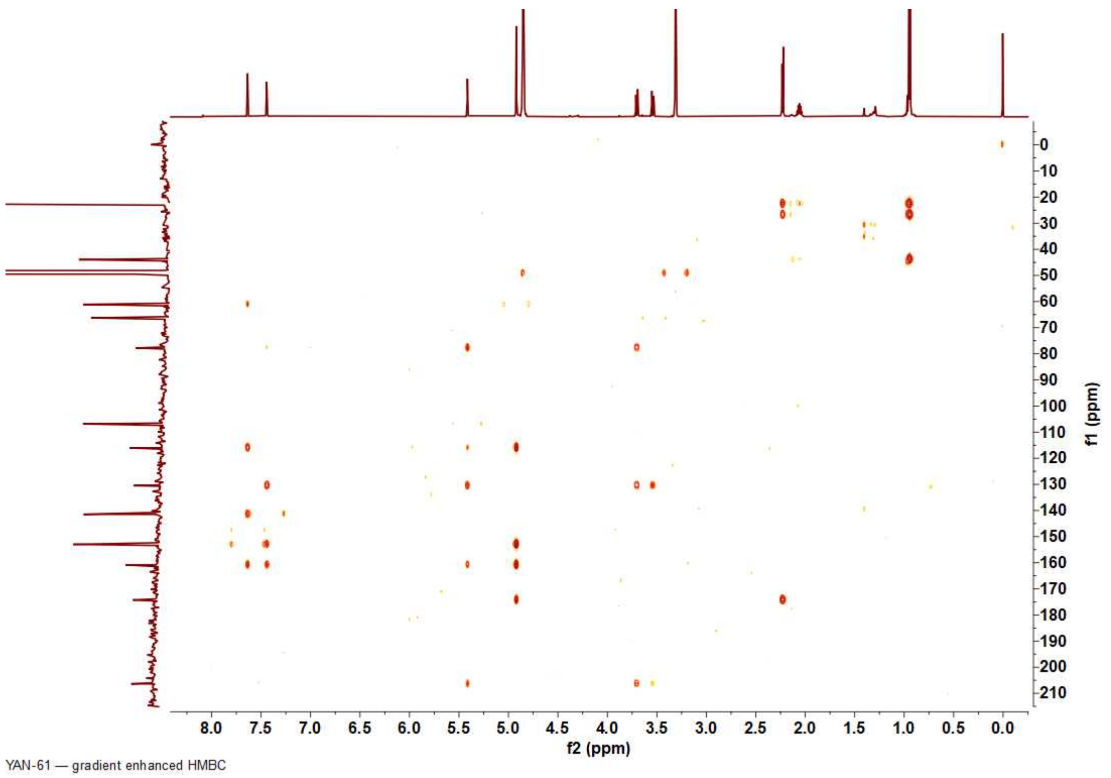

Figure S10. HMBC spectrum of 2 in CD_3OD

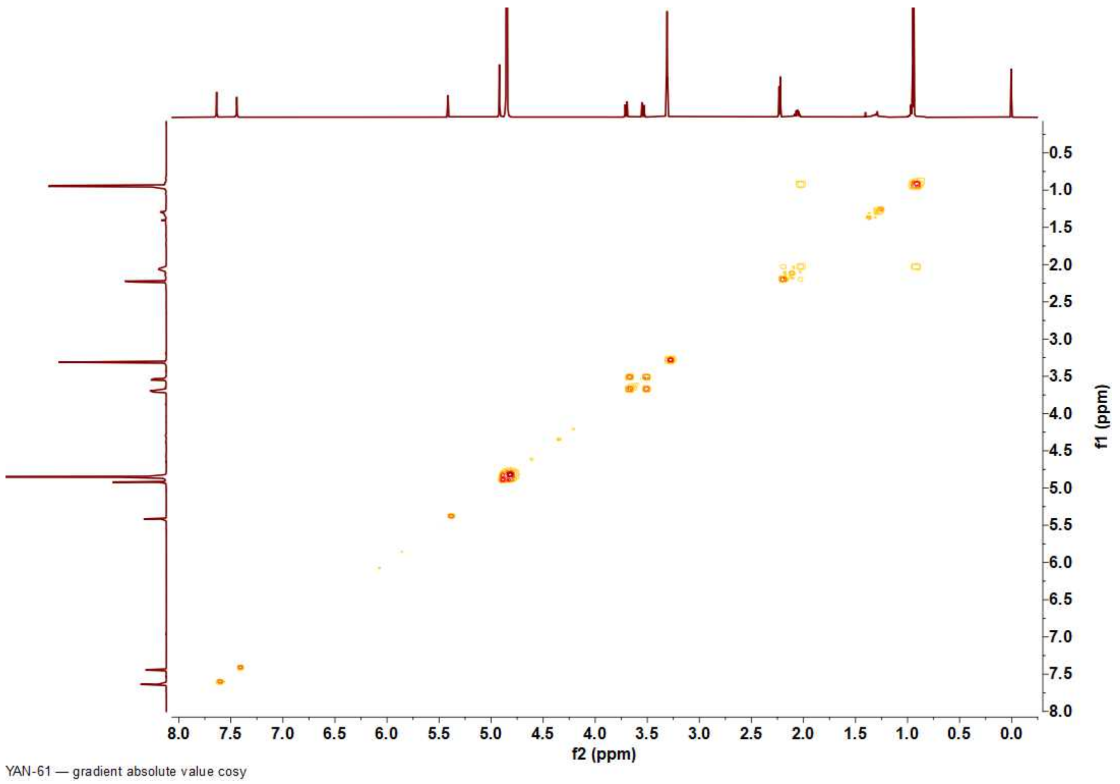

Figure S11. ^{1}H-^{1}H COSY spectrum of 2 in CD_3OD

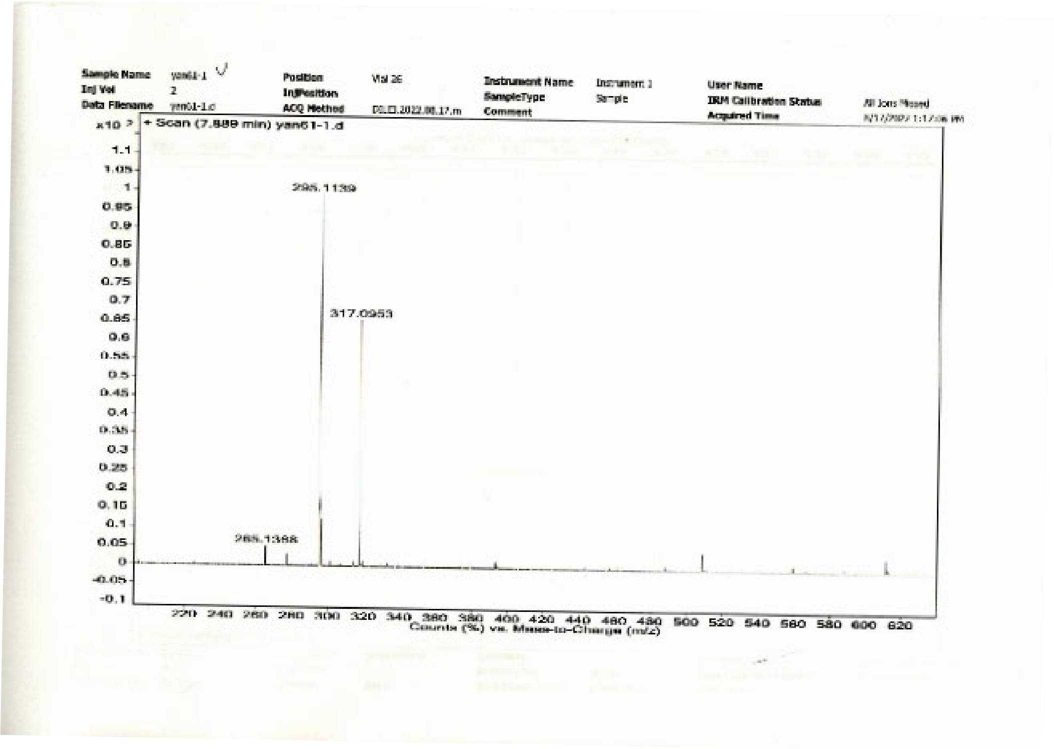

Figure S12. HRESIMS of 2

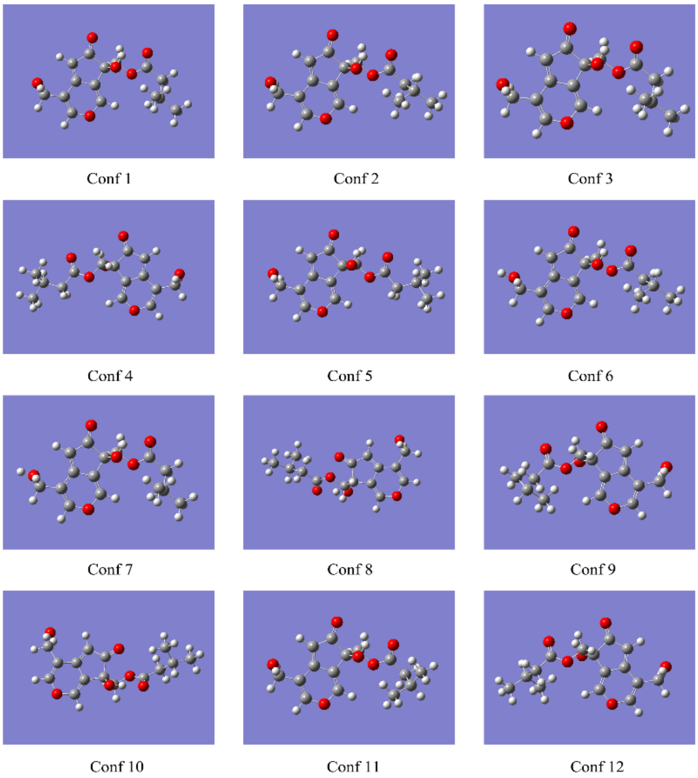

Figures S13 Optimized geometries of predominant conformers for 1

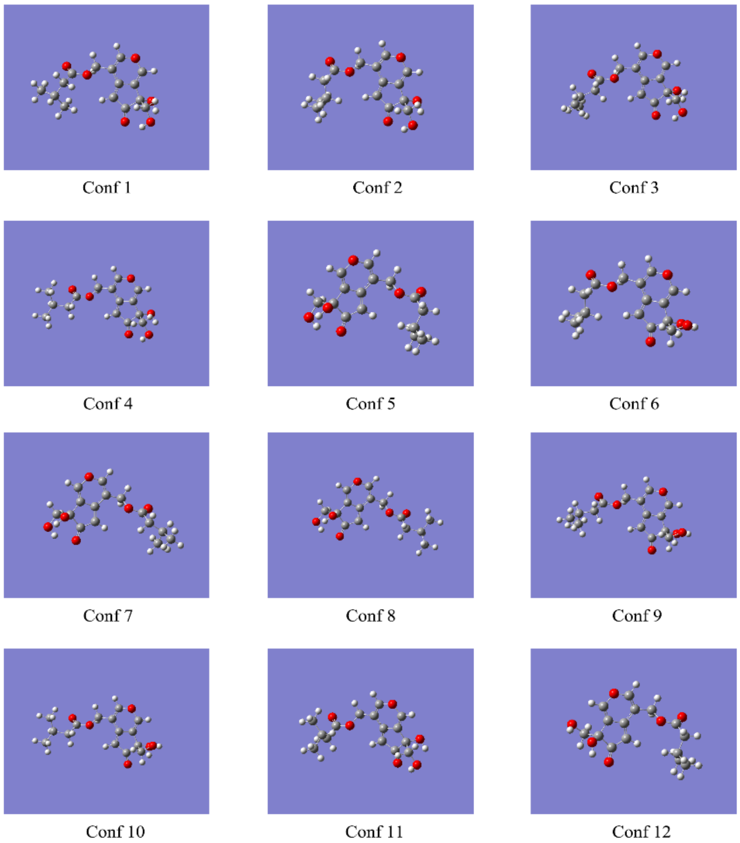

Figures S14 Optimized geometries of predominant conformers for 2

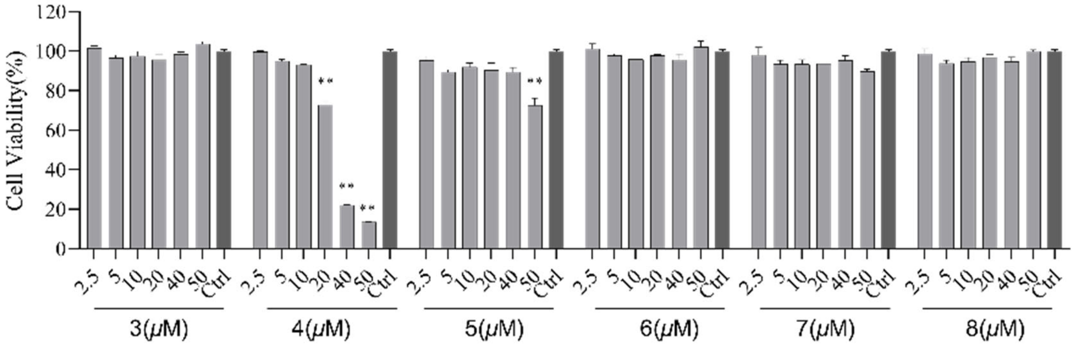

Figures S15: NRK-49f cell proliferation in response to compounds 3-8. Data represent mean ± SEM values of three experiments. * $p < 0.05$, ** $p < 0.01$ compared with Ctrl alone.

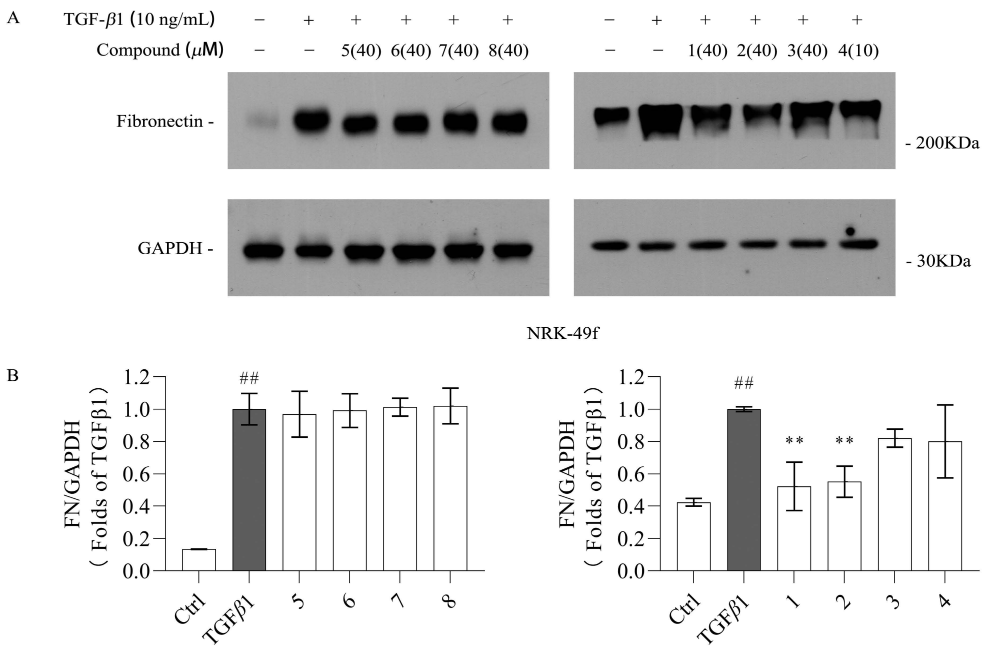

Figures S16: Effect of 1-8 on the expression of fibronectin in TGFβ1 induced NRK-49f.
